# Knowledge, Attitudes, and Practices of School Teachers Regarding Type 1 Diabetes Mellitus in Children in the United Arab Emirates

**DOI:** 10.7759/cureus.109911

**Published:** 2026-05-30

**Authors:** Humam Jamal, Soundharya Venkatesan, Raghad Muaiyad, Mohamed Ibrahim, Albatole Imad, Sharifa Salem, Amal Hussein

**Affiliations:** 1 Medicine, University of Sharjah, Sharjah, ARE; 2 Family and Community Medicine and Behavioral Sciences/Public Health, University of Sharjah, Sharjah, ARE

**Keywords:** childhood diabetes, knowledge-attitude-practice (kap), school teachers, type 1 diabetes mellitus, uae education system

## Abstract

Background

Type 1 diabetes mellitus (T1DM) is a common childhood condition requiring proper management, with teachers playing a vital role in recognizing symptoms and providing support. This study assesses their knowledge, attitudes, and practices to identify gaps and recommend improvements.

Aim

The study aims to assess school teachers' knowledge, attitudes, and practices regarding the management of T1DM in children in the UAE.

Methods

A descriptive cross-sectional study was conducted among 402 primary school teachers using convenience sampling. A structured, adapted 47-item questionnaire was developed and piloted. Data were analyzed using SPSS Version 25 (IBM SPSS Statistics for Windows, IBM Corp., Armonk, NY) with descriptive statistics and chi-square tests (p ≤ 0.05), and multivariable logistic regression was performed. Results were presented using bar and pie charts.

Results

Participants were predominantly female (293, 72.9%). A total of 277 (69.1%) participants were aged 31-50 years. Good knowledge was highest for questions such as blood glucose level determining dose of treatment (81.6%) and first-aid response when students felt unwell (80.8%), but lower for exercise precautions (52.7%) and hypoglycemia management (56.0%). Positive attitudes were widespread, with 91.6% strongly supporting school-based diabetes initiatives. Practices varied; while most teachers regularly observed blood glucose checks and insulin administration, only 38 of the experienced teachers (21.3%) had received formal training, and 116 (65.2%) followed standardized protocols. Knowledge was significantly associated with age, gender, family history of chronic illnesses and T1DM, scientific major, and prior experience with diabetic students (p < 0.05), while attitudes were mainly associated with knowledge and good practices (p < 0.001).

Conclusion

The study shows that the teachers’ willingness to support T1DM students exceeds their preparedness to manage care and emergencies. Establishing a unified, tiered-training and algorithm-based action plan, adapted from successful models by the ADA, across all UAE schools may potentially provide structured, step-wise instructions for routine and emergency care. Such a system may possibly enhance safety, reduce variability in practice, and ensure consistent, evidence-based support for students with T1DM.

## Introduction

Type 1 diabetes mellitus (T1DM) is a chronic metabolic disorder characterized by hyperglycemia due to insulin deficiency. T1DM is not characterized by insulin resistance and obesity; rather, it is an autoimmune disorder where the body's immune system attacks the insulin-producing β cells in the pancreas. However, type 2 diabetes mellitus (T2DM) is strongly linked to insulin resistance due to reasons like poor lifestyle and obesity. It can be managed with oral medications and lifestyle modifications. Insulin may be added later. While T1DM can onset at any age, it is typically diagnosed in childhood or adolescence. The lack of insulin production necessitates lifelong replacement therapy. There are many misconceptions about the difference between T1DM and T2DM, often causing delays in diagnosis, stigma, and false beliefs about their causes or management [1-3].

T1DM is one of the most common chronic endocrine diseases encountered in children and young people around the world, requiring continuous medical and psycho-social support to reduce the risk of complications like hypoglycemia and diabetic ketoacidosis, and long-term microvascular and macrovascular complications [4,5].

An estimated 79,074 individuals in the United Arab Emirates (UAE) live with T1DM, including approximately 9,939 children under the age of 20 years [6]. However, the absence of a unified national diabetes registry limits the accuracy of age-specific prevalence and incidence data, and existing estimates must therefore be interpreted with caution [7]. Most available data originate from hospital-based or regional studies, leading to considerable variability in reported disease burden and likely underestimation of true prevalence. These findings underscore the urgent need for stronger epidemiological surveillance and improved support systems for children with T1DM in the UAE.

Childhood and adolescence represent critical periods of physical, cognitive, and emotional development. Living with a chronic condition such as T1DM can adversely affect academic performance, school attendance, and psycho-social well-being if not adequately supported [8]. To acknowledge the diverse developmental stages of students, this study focuses on kindergarten, primary, middle, and high school students. In these earlier stages, teachers significantly impact students’ overall well-being. Kindergarten teachers lay the health literacy foundation, primary school teachers support holistic development, and secondary school and high school teachers direct students toward independence. The key component of this developmental approach is age-appropriate health literacy.

Children with T1DM depend on caregivers and school personnel for daily diabetes management, including blood glucose monitoring, insulin administration, dietary regulation, and timely recognition of hypoglycemia and hyperglycemia [9,10]. Focusing on these stages of development provides insights into student-teacher relationship dynamics and how teachers can nurture these children in developing self-health literacy and self-management skills, along with academic excellence. According to the American Diabetes Association, health literacy, for children and adolescents with T1DM who require ongoing support from teachers and caregivers, encompasses being able to understand, discuss, and apply basic health information about diabetes, such as recognizing signs, blood glucose testing, insulin, and nutrition, with adults to make safe health decisions appropriate to their stage of development. Additionally, children spend one-third to one-half of their waking hours in school. Hence, teachers play a pivotal role in ensuring a safe, inclusive, and supportive learning environment for students with T1DM [11,12].

Aim

Primary Objective

This study aims to assess the knowledge, attitudes, and practices (KAP) of school teachers regarding the management of T1DM in children across schools in the UAE.

Secondary Objective

The second objective of this study is to identify socio-demographic and professional factors associated with the KAP levels, evaluate the level of teachers' preparedness in diabetes care in the school setting, and assess their willingness to undergo diabetes-related training and provide student support.

## Materials and methods

Research design and sampling methods

This descriptive cross-sectional study was conducted in the UAE from September 2023 to June 2025. The data collection was conducted in two phases: the first from September 2023 to July 2024, and the second from May to June 2025, with additional participants. The study adhered to the principles of the Declaration of Helsinki [13]. Approval from the Research Ethics Committee of the University of Sharjah (approval number: REC-24-01-25-08-S) was obtained to conduct the study.

The minimum sample size was calculated by the single population proportion formula:

$$n = \frac{Z^2 p(1-p)}{d^2}$$

Applying a 95% confidence interval (Z = 1.96), 5% margin of error, and 63.3% expected prevalence into the formula, from a previous KAP reference study, yielded about 357 target participants [14]. The target sample size was increased to 393 participants in order to include a 10% possible non-response rate. A total of 402 complete responses were finally obtained and were included in the analysis.

Approval to collect data was obtained from the concerned public and private school authorities across the participating emirates. Convenience sampling was adopted due to feasibility and accessibility. 

Inclusion Criteria

The participants were teaching and non-teaching staff from both public and private schools. Participants over 20 years and teachers with any years of experience were included. Participants were from primary, intermediate, and secondary schools, of both genders, all majors, and from all the Emirates in the UAE. 

Exclusion Criteria

Participants who did not complete the questionnaire or answered the questions improperly were excluded from the study. Participants who declined informed consent, duplicate responses, substantial missing data, and pilot study participants were excluded. Participants less than 20 years old and not working within the UAE school system at the time of data collection were not a part of the study. 

Data collection

Data were collected using a modified version of a previously validated, 47-item questionnaire [14].

During the study period, trained research assistants administered the questionnaire to the principals and head teachers of multiple schools at different time points. The questionnaire was distributed in person, through email, and via social media. The questionnaire link was shared with other teachers by the principal and head teachers in the schools. All teachers completed the questionnaire, and eligibility was ensured through pre-set exclusion criteria embedded in Google Forms (Google LLC, Mountain View, CA), restricting further participation where applicable. It also helped in grouping participants into inexperienced and experienced teachers, and the separation of the relevant sections.

Informed consent was obtained from all participants. Teachers were informed of the study objectives, assured of confidentiality, and participated voluntarily.

The questionnaire comprised five sections: (1) socio-demographic characteristics, (2) chronic health conditions, (3) knowledge, (4) attitudes, and (5) practices related to T1DM.

Instrument Adaptation

To ensure contextual relevance and clarity, minor modifications were made to the original questionnaire without compromising its validity or accuracy. The instrument was available in both English and Arabic and was administered electronically through a structured survey format. The Arabic questionnaire was reviewed by bilingual experts to ensure linguistic equivalence. The questionnaire was pilot-tested with 10 teachers to assess clarity and comprehension. Internal consistency was not assessed after questionnaire modification. However, an expert review, by two experts in the field of public health, was performed to assess content validity and contextual relevance. Necessary amendments were made based on their feedback. 

The knowledge items followed a true/false/I don't know format. Practice items followed a yes/no/not sure format. Attitude items followed a 5-point Likert scale. The attitudes and practices section of the questionnaire assessed the willingness of teachers to undergo diabetes care-related training and their perceived preparedness to support students with T1DM. Both included hypothetical and scenario-based questions like confidence in handling hypoglycemia, willingness to receive training, and ability to support insulin or glucose monitoring in school settings or classrooms.

The full questionnaire is provided in Appendix 1.

Statistical analysis

Data analysis was performed using SPSS Version 25 (IBM SPSS Statistics for Windows, IBM Corp., Armonk, NY). A complete case analysis was performed. Descriptive statistics (frequencies and percentages) summarized socio-demographic characteristics and KAP scores.

Correct knowledge and practice responses were scored as one point, while incorrect or uncertain responses scored zero. Attitudes were scored 0-5 points. Scores below 60% were classified as poor knowledge, attitudes, or practices. A commonly used cutoff of 60% was adopted from previous studies in the healthcare and educational settings, where >60% was considered adequate knowledge/practices/attitudes, allowing easy comparability between similar studies [15-17]. 

Reverse coding was applied to negatively worded items. Bivariate analysis was performed using cross-tabulations with chi-square tests to examine associations between socio-demographic variables and KAP levels. Results were illustrated using bar graphs and pie charts.

Independent predictors of good knowledge and practices of inexperienced and experienced teachers regarding T1DM were analyzed using multivariable logistic regression analysis. Selection of the variables in the regression models was based on theoretical relevance and bivariate analysis findings. Adjusted odds ratios (95% confidence interval) were reported. The Hosmer-Lemeshow goodness-of-fit test, omnibus tests of model coefficients, Nagelkerke R² values, and classification accuracy were used to assess the fitness of the overall models. p ≤ 0.05 was taken as statistically significant.

## Results

Socio-demographic characteristics and health information of school teachers

A total of 402 participants took part in the study. Socio-demographic characteristics and health information of school teachers are summarized in Table 1.

**Table 1:** Socio-demographic characteristics and health information of school teachers T1DM - type 1 diabetes mellitus

Characterization	N (%)
Gender	
Male	109 (27.1%)
Female	293 (72.9%)
Age (years)	
20-30	61 (15.2%)
31-40	122 (30.3%)
41-50	155 (38.6%)
>50	64 (15.9%)
Field of study	
Scientific	97 (24.1%)
Non-scientific	305 (75.9%)
Employment type	
Teacher	344 (85.6%)
Non-teacher	58 (14.4%)
Years of experience	
0-5	88 (21.9%)
6-10	76 (18.9%)
11-15	74 (18.4%)
16-20	62 (15.4%)
>20	102 (25.4%)
Education sector	
Kindergarten	30 (7.5%)
Elementary	95 (23.6%)
Middle	73 (18.2%)
High school	204 (50.7%)
Emirate	
Sharjah	218 (54.2%)
Dubai	43 (10.7%)
Abu Dhabi	89 (22.1%)
Ras Al Khaimah	12 (3.0%)
Ajman	24 (6.0%)
Fujairah	10 (2.5%)
Um Al Quwain	6 (1.5%)
Type of school	
Public	160 (39.8%)
Private	242 (60.2%)
Education level	
Undergraduate	313 (77.9%)
Postgraduate	89 (22.1%)
Family history of T1DM	
Yes	140 (34.8%)
No	237 (59.0%)
I don’t know	25 (6.2%)
Family history of chronic health conditions	
Yes	211 (52.5%)
No	176 (43.8%)
I don’t know	15 (3.7%)

As shown in Table 1, 293 (72.9%) participants were female, and about 277 (68.9%) were between 31 and 50 years. About 344 (85.6%) were teachers, while 58 (14.4%) were non-teaching staff, and 376 (93.5%) participants held a degree.

The years of teaching experience varied among participants: 88 (21.9%) participants had zero to five years of teaching experience, 76 (18.9%) had six to 10 years, 74 (18.4%) had 11-15 years of experience, 62 (15.4%) had 16-20 years, and 102 (25.4%) had more than 20 years of experience. Regarding level of education, half of the participants taught high school, about 204 (50.7%).

Participants were distributed across all the Emirates: 218 (54.2%) of participants in Sharjah, 43 (10.7%) in Dubai, 89 (22.1%) in Abu Dhabi, 12 (3.0%) in Ras Al Khaimah, 24 (6%) in Ajman, 10 (2.5%) in Fujairah, and 6 (1.5%) in Umm Al Quwain.

Over 140 (34.8%) participants had T1DM in their families, while 211 (52.5%) had chronic health conditions in their families.

Understanding of T1DM 

Knowledge Levels Across Various Socio-Demographic Characteristics

Levels of knowledge differed across various socio-demographic factors. A higher knowledge score was observed among older participants, with the highest being in the group aged >50 years (68.75%), while the lowest was in the 20-30 year-old group (37.70%), as described in Table 2.

**Table 2:** Proportion of teachers with good knowledge across socio-demographic factors Practices (taught): Practices of teachers who taught students with type 1 diabetes mellitus. Practices (non-taught): Practices of teachers who did not teach students with type 1 diabetes mellitus. T1DM - type 1 diabetes mellitus

Variable	Category	% Good knowledge	Statistical test	p-value (p < 0.05)
Age	20-30	37.70%	Chi-square	0.003
	31-40	59.83%	-	-
	41-50	61.29%	-	-
	>50	68.75%	-	-
Gender	Male	48.62%	Chi-square	0.015
	Female	62.12%	-	-
Qualifications	Diploma	46.15%	Chi-square	0.323
	Bachelors	60.97%	-	-
	Masters	52.63%	-	-
	PhD	61.53%	-	-
Family history	Yes	72.86%	Chi-square	<0.001
	No	53.16%	-	-
	I don’t know	28.00%	-	-
Type of school	Public	58.13%	Chi-square	0.720
	Private	57.85%	-	-
Field of study	Scientific	74.22%	Chi-square	<0.001
	Non-scientific	53.44%	-	-
Have you taught a student with T1DM? (prior exposure to students with T1DM)	Yes	75.28%	Chi-square	<0.001
	No	53.21%	-	-
	Not sure	26.47%	-	-
Years of experience	0-5	47.73%	Chi-square	0.020
	6-10	61.84%	-	-
	11-15	55.41%	-	-
	16-20	53.23%	-	-
	>20	70.59%	-	-
Education sector	Kindergarten	46.67%	Chi-square	0.388
	Elementary	54.74%	-	-
	Middle	60.27%	-	-
	High school	61.27%	-	-
Practices (taught)	Good	77.17%	Chi-square	0.423
Practices (non-taught)	Good	53.24%	Chi-square	<0.001

From Table 2, a higher level of knowledge was seen in females compared to males (62.12% vs. 48.62%), with a significant association between knowledge levels and gender (p = 0.015). The knowledge levels were highest in participants whose family members had T1DM (75.28%) compared to those who did not have a family history (53.16%) and those who were not sure (28%) (p < 0.001).

There was a significant association, where a higher level of knowledge was observed among the participants with scientific specialization (74.22%) compared to non-scientific specialization (53.44%) (p < 0.001). A higher level of knowledge was observed among participants with more years of experience (70.59%), and the lowest among those with up to five years of teaching experience (47.73%) (p = 0.020). Those with prior exposure to students with T1DM showed higher knowledge (75.28%) than those without prior exposure (53.21%) or those unsure (26.47%) (p < 0.001).

There was no significant association between the type of school, qualification, or sector of education and knowledge levels. 

Knowledge of Disease Characteristics and Management 

A total of 269 (66.9%) teachers accurately identified T1DM as a chronic condition. About 325 (80.8%) participants correctly addressed the need for continuous monitoring. Their knowledge about the age of onset was moderate, since only 198 (49.3%) of them answered the question correctly. Misconceptions existed when answering the causes of T1DM, in which 169 (42.0%) of them perceived the cause to be linked to lifestyle. Their knowledge about complications was high, with 319 (79.4%) participants answering it correctly. Questions about emergencies, such as hypoglycemic episode initial step and prompt management, were correctly addressed by 325 (80.8%) and 225 (56.0%) participants, respectively, as described in Table 3.

**Table 3:** Responses to knowledge questions among teachers T1DM - type 1 diabetes mellitus

Knowledge questions of the school teachers (n = 402)	True N (%)	False N (%)	I don't know N (%)
T1DM is diagnosed most frequently during childhood.	198 (49.3%)	63 (15.7)	141 (35.0%)
T1DM is a chronic disease.	269 (66.9%)	20 (4.9%)	113 (28.2%)
T1DM is caused by poor lifestyle such as lack of physical activity, unhealthy food, pollution, etc.	169 (42.0%)	130 (32.3%)	103 (25.7%)
T1DM can have serious complications if not treated appropriately.	319 (79.4%)	11 (2.7%)	72 (17.9%)
People with T1DM are advised to regularly measure the level of sugar (glucose in their blood).	325 (80.8%)	12 (3.0%)	65 (16.2%)
The normal blood sugar level in children upon awaking is 4-7 mmol/L or equivalent to 72-120 mg/dL.	167 (41.5%)	19 (4.7%)	216 (53.7%)
The level of sugar in the blood is important to determine the dose of treatment to be taken.	328 (81.6%)	11 (2.7%)	63 (15.7%)
If blood sugar levels are normal for 3 months diabetes medications can be stopped.	86 (21.4%)	138 (34.3%)	178 (44.3%)
People with T1DM only use insulin when complications occur.	115 (28.6%)	118 (29.4%)	169 (42.0%)
Insulin use carries a risk of (low blood sugar) hypoglycemic episode.	182 (45.5%)	38 (9.5%)	181 (45.0%)
People with T1DM must eat more carbohydrates (sugars) because their bodies do not produce the sugar needed for energy.	79 (19.7%)	174 (43.3%)	149 (37.1%)
Red meat is a high-carb food.	89 (22.1%)	220 (54.7%)	93 (23.2%)
People who take diabetes medications don't need to care about healthy eating.	53 (13.2%)	299 (74.4%)	50 (12.4%)
Injuries to the skin are more serious in people with T1DM because the risk of infection is higher.	275 (68.4%)	29 (7.2%)	98 (24.4%)
Intense exercise may cause severe (low blood sugar) hypoglycemia in patients with T1DM.	235 (58.5%)	39 (9.7%)	128 (31.8%)
There are no warning signs of (low blood sugar) hypoglycemia.	68 (16.9%)	240 (59.7%)	94 (23.4%)
People with T1DM need regular medical exams for their eyesight, kidney function, and nerve function.	300 (74.6%)	18 (4.5%)	84 (20.9%)
There are no specific measures/precautions for people with T1DM to exercise.	78 (19.4%)	212 (52.7%)	112 (27.9%)
If a person with T1DM feels unwell, the first thing to do is check their blood sugar level.	325 (80.8%)	15 (3.7%)	62 (15.4%)
If a person with T1DM has an episode of hypoglycemia, they should eat sugary foods or drinks.	225 (56.0%)	58 (14.4%)	119 (29.6%)

**Figure 1:**
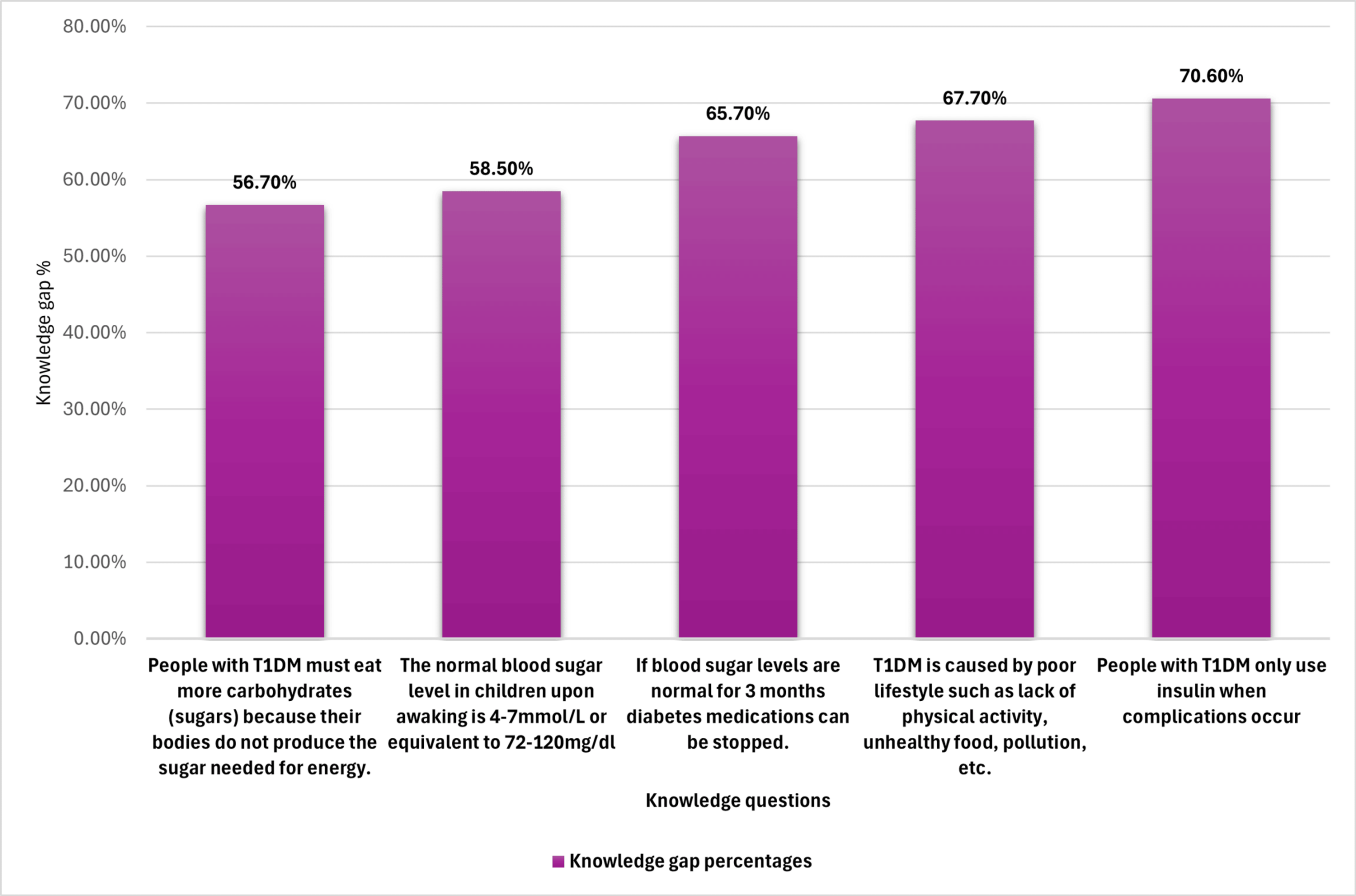
Significant knowledge gaps of school teachers (>55%) T1DM - type 1 diabetes mellitus

A knowledge gap of more than 55% was considered a substantial knowledge gap. Many participants perceived T1DM to be a lifestyle disorder (67.70%) and thought insulin was only used if the child had a complication (70.60%). They also misconceived that diabetes medications can be stopped once blood sugar levels are normal for three months (65.70%). Significant knowledge gap values of more than 55% are presented in Figure 1.

The detailed assessment for knowledge gaps among school teachers regarding students with T1DM is presented in Appendix 2.

Practices and attitudes of teachers in managing children with T1DM

A total of 178 teachers have taught students with T1DM. The detailed assessment of practices of teachers who taught students with T1DM (practices of experienced teachers) is presented in Table 4.

**Table 4:** Practices of teachers who taught children with type 1 diabetes mellitus

Practices of teachers who taught children with T1DM (n = 178)	Yes n (%)	No n (%)	Not sure n (%)
Have you actively implemented specific measures to ensure the safety and well-being of a diabetic student during regular class hours?	114 (64.0%)	45 (25.3%)	19 (10.7%)
In emergency situations related to a student with type 1 diabetes's health, have you followed specific protocols or procedures?	116 (65.2%)	32 (18.0%)	30 (16.8%)
Do you regularly check in with students with type 1 diabetes to ensure they are following their care plan, including checking blood sugar levels or administering insulin if needed?	92 (51.7%)	71 (39.9%)	15 (8.4%)
Have you received specific training or professional development related to supporting students with type 1 diabetes in your classroom?	38 (21.3%)	128 (71.9%)	12 (6.7%)
Are you comfortable discussing and addressing the specific needs of a student with type 1 diabetes with their parents or caregivers?	143 (80.3%)	17 (9.6%)	18 (10.1%)

As shown in Table 4, among the experienced teachers, only 38 (21.3%) had formal diabetes-related training. Safety measures and emergency protocols were implemented by 114 (64.0%) and 116 (65.2%) experienced teachers, respectively. About 143 (80.3%) teachers with experience felt comfortable discussing with parents or addressing diabetes-related needs.

The responses in Table 5 were taken separately from participants who have not taught students with T1DM (inexperienced teachers), as their lack of experience makes it difficult to answer questions on practices. Hence, questions were tailored to assess their practices and their willingness in various hypothetical scenarios similar to the practice questions that assessed experienced teachers.

**Table 5:** Frequency distribution of practices among teachers who have not encountered students with type 1 diabetes mellitus T1DM - type 1 diabetes mellitus

Practices of teachers who have not encountered students with T1DM (n = 224 )	Yes n (%)	No n (%)	Not sure n (%)
If you have a student with type 1 diabetes in your classroom, do you feel confident in your ability to implement specific measures to ensure their safety and well-being during regular class hours?	100 (44.6%)	48 (21.4%)	76 (33.9%)
If you were to have a student with type 1 diabetes in your classroom, would you proactively seek information about their specific needs and care requirements?	176 (78.6%)	15 (6.7%)	33 (14.7%)
In a hypothetical scenario where a student with type 1 diabetes needed to check their blood sugar level or administer insulin during class, do you think you would handle the situation effectively?	126 (56.3%)	41 (18.3%)	57 (25.4%)
Considering you haven't encountered a student with type 1 diabetes, would you be willing to undergo training or seek guidance on how to support and accommodate their needs in the classroom?	164 (73.2%)	15 (6.7%)	45 (20.1%)
If you were aware that a student in your class has type 1 diabetes, would you make an effort to communicate with their parents or caregivers to stay informed about their health and care plan?	176 (78.6%)	11 (4.9%)	37 (16.5%)

According to Table 5, when asked hypothetically, only 100 (44.6%) of inexperienced teachers were confident about implementing safety measures, and 126 (56.3%) of them were confident about handling emergencies in the classroom or school settings. About 176 (78.6%) of inexperienced teachers admitted that they would proactively seek information about T1DM, and 164 (73.2%) were willing to undergo training.

Attitudes of both experienced and inexperienced teachers were assessed separately, which showed all of them exhibiting positive attitudes, approximately 86-88% agreeing that training, emergency help, and student support are their essential duties, and only 1-3% strongly disagreed with the statements, as shown in Table 6.

**Table 6:** Frequency distribution of attitude-related responses among all school teachers T1DM - type 1 diabetes mellitus

Attitudes of all teachers (n = 402)	Strongly disagree + disagree n (%)	Neutral n (%)	Strongly agree + agree n (%)
Do you support starting a project to improve diabetes control during school hours?	3 (0.7%)	31 (7.7%)	368 (91.6 %)
Are you open to getting training from professionals on how to support children with T1DM at school?	11 (2.7%)	42 (10.4%)	349 (86.9%)
Would you be willing to learn how to help a student with T1DM who isn't feeling well?	7 (1.7%)	39 (9.7%)	356 (88.6%)
Are you willing to assist a student with T1DM until an ambulance or specialist care arrives?	9 (2.2%)	49 (12.2%)	344 (85.6%)
Are you willing to receive training from professionals regarding school measures for students with other chronic diseases?	5 (1.3%)	47 (11.7%)	350 (87%)

Bivariate analysis of the association between practices of experienced teachers and knowledge, performed by chi-square statistics, did not show a significant association, which was also confirmed by multivariate logistic regression, which showed an insignificant association, after adjusting for confounders (OR =1.30, 95% CI: 0.52-3.26, p = 0.576). 

Experienced teachers with good attitudes demonstrated a higher proportion of good practices (p = 0.009). 

In contrast, practices of inexperienced teachers were significantly associated with both knowledge and attitudes (both p < 0.001). 

A higher proportion of good practices was seen in inexperienced teachers with good knowledge (89.1%) (p < 0.001) and good attitudes (77.7%) (p < 0.001). 

Practices of inexperienced teachers were significantly associated with the presence of a family history of chronic illnesses (p = 0.013), with higher practice levels reported among teachers with positive family history. In contrast, no significant association was observed between family history of chronic illnesses and practices of experienced teachers (p = 0.734).

Also, attitudes were significantly associated with knowledge (p < 0.001). Teachers with good knowledge had higher rates of good attitudes (95.3%) as compared to those with poor knowledge (85.0%) (p < 0.001).

Attitudes were significantly associated with practices of inexperienced and experienced teachers, with a significance of (p < 0.001) and (p = 0.025), respectively.

Complete bivariate analysis of practices and attitudes of teachers with various variables is provided in Appendix 3 and Appendix 4.

Multivariable logistic regression analysis of factors associated with knowledge and practices

Multivariable logistic regression analysis was conducted to predict independent predictors of good knowledge among teachers. The model contained socio-demographic, educational, family history, and prior teaching exposure variables. After adjustment, prior teaching exposure to students with T1DM, female gender, teachers aged 31-40 years, 41-50 years, having a scientific specialization, a positive family history of T1DM, and the presence of chronic diseases were significantly associated with good knowledge (p < 0.05). School type and years of experience were not significant, as shown in Table 7.

**Table 7:** Multivariable logistic regression analysis of factors associated with good knowledge among teachers (n = 402) Nagelkerke R² = 0.295; Hosmer-Lemeshow test: χ² = 9.461; p = 0.305 (good fit); -2 log likelihood = 446.199, Cox-Snell R² = 0.219. Reference categories: age (20-30 years), gender (male), major (non-scientific specialization), type of school (public), year of teaching experience (zero to five years), family history of T1DM (no), family history of chronic disease (no), and prior teaching exposure to students with T1DM (no). T1DM - type 1 diabetes mellitus

Variable	p-value	Adjusted OR	95% CI
Age (31-40 years)	0.008	2.909	1.323-6.398
Age (41-50 years)	0.007	3.463	1.401-8.560
Age (>50 years)	0.061	3.243	0.947-11.113
Female gender	0.003	2.359	1.331-4.181
Scientific specialization	0.009	2.174	1.216-3.889
Years of teaching experience	0.39	-	-
Type of school (private)	0.692	1.11	0.663-1.859
Family history of T1DM (yes)	0.004	2.159	1.281-3.638
Family history of chronic disease (yes)	0.039	1.678	1.027-2.739
Prior teaching exposure to students with T1DM (yes)	<0.001	3.885	2.352-6.418

A multivariable logistic regression analysis was conducted to determine the factors associated with good practice among experienced teachers. The model comprised knowledge, attitude score, and selected socio-demographic variables. In the adjusted model, none of the socio-demographic variables, including age, gender, scientific specialization, and type of school, were statistically significantly associated with good practice (all p > 0.05). Even knowledge was not significantly related to good practice levels (p = 0.576). The attitude score showed a positive trend toward better practice, but this association was not statistically significant (p = 0.063). No independent predictors of good practice were observed in the final adjusted model, as shown in Table 8.

**Table 8:** Multivariable logistic regression analysis of factors associated with good practice in experienced teachers (n = 178) Nagelkerke R² = 0.142; Hosmer-Lemeshow test: χ² = 3.924; p = 0.864 (good fit); -2 log likelihood = 214.142; Cox-Snell R² = 0.104. Reference categories: age (20-30 years), gender (male), major (non-scientific specialization), type of school (public), knowledge (poor), and attitude (poor).

Variable	p-value	Adjusted OR	95% CI
Good knowledge	0.576	1.30	0.52-3.26
Age (31-40 years)	0.638	0.73	0.20-2.66
Age (41-50 years)	0.944	0.95	0.22-4.02
Age (>50 years)	0.798	0.79	0.13-4.67
Female gender	0.863	0.93	0.42-2.08
Scientific specialization	0.737	0.88	0.40-1.91
Type of school (private)	0.256	0.66	0.32-1.36
Good attitude	0.063	3.76	0.93-15.16

A multivariable logistic regression analysis determined the factors associated with good practice among inexperienced teachers. The model included knowledge, attitude score, and selected demographic variables. In inexperienced teachers, good knowledge and positive attitude were strong predictors of good practice. Female gender, teachers aged 31-40 years, and those working in elementary schools and high schools were also significantly associated with better practice. Other variables in the model were not statistically significant, as shown in Table 9.

**Table 9:** Multivariable logistic regression analysis of factors associated with good practice in inexperienced teachers (n = 224) Nagelkerke R² = 0.402; Hosmer-Lemeshow test: χ² = 6.681; p = 0.571 (good fit); -2 log likelihood = 186.134; Cox-Snell R² = 0.275. Reference categories: age (20-30 years), gender (male), knowledge (poor), attitude (poor), and education sector (kindergarten).

Variable	p-value	Adjusted OR	95% CI
Good knowledge	0.001	4.738	1.824-12.306
Good attitude	<0.001	10.826	3.309-35.423
Age (31-40 years)	0.041	3.858	1.056-14.089
Female gender	0.037	2.950	1.069-8.144
Elementary sector	0.045	3.844	1.033-14.299
High school sector	0.001	10.064	2.528-40.063

## Discussion

This study assesses KAP of school teachers toward T1DM in school children, including socio-demographic and professional factors associated with their KAP scores. Various studies assessing KAP of school teachers from other Middle Eastern regions were referred to derive interpretations and conclusions [18-23]. It is essential to understand teachers’ preparedness as students spend a significant portion of their day in schools and might require daily diabetic care or management of glycemic emergencies. The study is consistent with reports from other Middle Eastern and international studies showing a significant gap in teachers’ knowledge about T1DM and practical competencies despite positive attitudes.

Knowledge levels and determinants

Knowledge levels, when associated with socio-demographic characteristics, described that teachers' knowledge was correlated to their education acquired, previous personal and familial experiences, and exposure. It was not associated with institutional factors like type of school, qualification levels, or the sector of education. Higher knowledge levels were observed among older participants, females, and those with scientific backgrounds and prior exposure to students with T1DM. However, these results should be interpreted cautiously due to the cross-sectional design and potential residual confounding.

Bivariate analysis showed that higher T1DM knowledge was associated with increased age, years of teaching experience, a scientific background, prior family history, and teaching exposure to T1DM. Strong associations were found between exposure and training (p < 0.001), suggesting that knowledge is correlated with personal and professional experience. These findings reflect associations rather than causation.

Attitudes toward supporting students with T1DM

Both experienced and inexperienced teachers exhibited positive attitudes toward supporting students with T1DM, expressing a strong sense of responsibility and willingness to help. This provides a foundation for developing an inclusive and supportive school environment. Nevertheless, positive attitudes alone did not ensure preparedness because many teachers showed limited confidence in managing diabetes-related situations, like implementation of specific measures to ensure the safety of students with T1DM (Table 4 and Table 5), assisting a student until emergency services arrive, and responding to a student who is unwell during class hours, including situations requiring glucose monitoring or insulin administration.

Practices, experience, and preparedness

A significant gap existed between teachers’ attitudes and their actual practices, despite their high willingness. Only a few teachers received formal training and showed inconsistent adherence to safety measures and emergency protocols. Experienced teachers reported greater confidence and comfort, mainly in communication with parents about the students’ condition. However, multivariate analysis showed no independent predictors of good practice among experienced teachers. This suggests observed associations may be influenced by residual confounding. Nevertheless, there was an overall low level of confidence, most likely associated with persistent dependence on personal experience rather than standardized school protocols. Due to the nature of the study design, these relationships cannot confirm directionality.

KAP relationships

Knowledge and attitudes were significantly associated with practices of inexperienced teachers, mostly correlating to theoretical understanding and perceptions shaping anticipated behaviors.

 In contrast, associations of practices with knowledge and attitudes were not significant among experienced teachers, possibly reflecting accumulated diabetes management practical exposure and familiarity rather than theoretical knowledge or personal beliefs. This interpretation remains speculative and should be viewed as hypothesis-generating.

Study limitations

The study provides insights into the KAP of school teachers regarding T1DM, and the findings should be interpreted accordingly. Being a cross-sectional study, only associations can be inferred, and causal relations cannot be established or interpreted longitudinally over time.

Use of a self-administered questionnaire may introduce recall and social desirability bias, despite the convenience of the sampling method to reach a large population. Most of the teachers were female due to a gender distribution in the profession. Generalizability is limited as most teachers were from Sharjah, although some were from other Emirates. Some subgroup analyses, such as for experienced teachers, involved smaller sample sizes, which statistically affect stability and precision of estimates.

The modified questionnaire was not retested for internal consistency using reliable measures such as Cronbach’s alpha after adaptation, limiting the psychometric robustness of the tool.

The substantial sample size provides insight into the existing diabetes care preparedness in schools. The study does not report practices through direct observation or assess the quality of training in trained individuals. The training is self-reported and not standardized. Details such as duration, quality, competency, and content of the training were not collected, limiting the interpretation of training effectiveness. This study does not assess the heterogeneity, quality, duration, and standardization of prior diabetes care-related training, which limits the interpretation of training-related findings and should be addressed in future studies.

Implications for school-based diabetes care

The results show the importance of hands-on experience in enhancing the effective management of diabetes within a school setting. All the similar studies referred to showed that teachers showed good attitudes but limited knowledge and practical experience to handle emergencies or provide required medical attention [18-23]. 

Children living with T1DM require special attention, which could be challenging for untrained staff in schools. Suggested levels of training, according to the American Diabetes Association, include the following:

Level 1 indicates basic diabetes awareness and symptoms for all people within schools. 

Level 2 indicates staff levels that directly work with diabetes patients and understand how to treat hypoglycemia and hyperglycemia [24].

Level 3 indicates that all teachers need not be trained, but for those who take on specific tasks, such as monitoring glucose levels, administering insulin, and administering glucagon, training and instruction are provided.

However, tiered training should be paired with an individualized care plan. Having an individualized Diabetes Medical Management Plan (DMMP) from the student’s healthcare provider provides specific guidance on managing glucose monitoring, insulin administration, and emergencies like hypoglycemia or hyperglycemia [24-26].

This study suggests the potential need for diabetes care preparedness training in schools in addition to teachers’ personal experience and perceptions.

## Conclusions

This study identifies a significant gap between knowledge and practical skills in school teachers regarding T1DM despite positive attitudes. Key findings of the study were teachers' inability to use insulin, recognize hypo- or hyperglycemia, and respond to emergencies. Additionally, teaching experience and prior exposure to diabetic students were associated with teachers’ knowledge and practices, emphasizing the importance of hands-on experience and targeted training. Thus, the findings highlight possible broader systemic issues such as school infrastructure, untrained school staff, and the absence of a unified standardized diabetes regulation and plan. This study creates scope for future research assessing the effectiveness of the current training programs and how the implementation of a standardized program may potentially improve the competency of teachers and the holistic progress of such students. Such strategies have been reported to improve teachers' confidence, as well as students' safety, even in global health communities.

The results of the study support the need for structured diabetes-care related tiered-training and DMMP for school settings. Such measures may support learning experience, academic growth, and student safety, while creating more uniformly inclusive, equitable, and prepared school environments for all children with T1DM across every school in the UAE.

## Appendices

Appendix 1

Questionnaire: Assessing Knowledge, Attitude, and Practice of School Teachers on Type 1 Diabetes Among Children in the UAE

1. Age Mark only one oval. ○ 20-30 ○ 31-40 ○ 41-50 ○ >50

2. Gender Mark only one oval. ○ Male ○ Female

3. Qualification Mark only one oval. ○ Diploma ○ Bachelor ○ Master ○ PhD

Major: ○ Mathematics ○ Science ○ Islamic studies ○ Art education ○ Social studies ○ Arabic language ○ English language ○ Physical education

Others specify __________

5. Professional title: ○ Principal ○ Deputy principal ○ Teacher ○ Student counselor

6. Type of school: ○ Public ○ Private

7. Years of experience ○ 0-5 ○ 6-10 ○ 11-15 ○ 16-20 ○ >20

8. Education sector: ○ Kindergarten ○ Elementary ○ Middle ○ High school

9. Emirates: ○ Sharjah ○ Dubai ○ Abu Dhabi ○ Ras Al Khaimah ○ Ajman ○ Fujairah ○ Umm Al Quwain

Family history of DM or other chronic diseases and experience of teacher with a diabetic student

10. Do you have a family history of type 1 diabetes mellitus (T1DM)? Mark only one oval. ○ Yes ○ No ○ I don’t know

11. Does anyone in your family have a history of long-term health issues? Mark only one oval. ○ Yes ○ No ○ I don’t know 

Knowledge: N) Kindly answer each statement below with “True”, “False”, OR “I do not know”. Note: T1DM is type 1 diabetes mellitus (T1DM).

1. T1DM is a long-lasting disease. ○ True ○ False ○ I don't know

2. T1DM is diagnosed most frequently during childhood. ○ True ○ False ○ I don't know

3. T1DM is caused by poor lifestyle, such as lack of physical activity, unhealthy food, pollution, etc. ○ True ○ False ○ I don't know

4. T1DM can have serious complications if not treated appropriately * ○ True ○ False ○ I don't know

5. People with T1DM are advised to regularly measure the level of sugar (glucose) in their blood ○ True ○ False ○ I don't know

6. The normal blood sugar level in children upon awakening is 4-7 mmol/L or equivalent to 72-120 mg/dL. ○ True ○ False ○ I don't know

7. The level of sugar in the blood is important to determine the dose of treatment to be taken Mark only one oval. ○ True ○ False ○ I don't know

8. If blood sugar levels are normal for 3 months, diabetes medications can be stopped. Mark only one oval. ○ True ○ False ○ I don't know

9. People with T1DM only use insulin when complications occur. ○ True ○ False ○ I don't know

10. Insulin use carries a risk of (low blood sugar) hypoglycemic episodes. * ○ True ○ False ○ I don't know

11. People with T1DM must eat more carbohydrates (sugars) because their bodies do not produce the sugar needed for energy. ○ True ○ False ○ I don't know

12. Red meat is a high-carb food. ○ True ○ False ○ I don't know

13. People who take diabetes medications don't need to care about healthy eating. ○ True ○ False ○ I don't know

14. Injuries to the skin are more serious in people with T1DM because the risk of infection is higher. ○ True ○ False ○ I don't know

15. Intense exercise may cause severe (low blood sugar) hypoglycemia in patients with T1DM. Mark only one oval. ○ True ○ False ○ I don't know

16. There are no warning signs of (low blood sugar) hypoglycemia. * ○ True ○ False ○ I don't know 

17. People with T1DM need regular medical exams for their eyesight, kidney function, and nerve function. ○ True ○ False ○ I don't know

18. There are no specific measures/precautions for people with T1DM to exercise. * ○ True ○ False ○ I don't know

19. If a person with T1DM feels unwell, the first thing to do is check their blood sugar level. * ○ True ○ False ○ I don't know

20. If a person with T1DM has an episode of hypoglycemia, they should eat sugary foods or drinks. ○ True ○ False ○ I don't know

Attitude: O) Please respond to the following questions by indicating the extent to which you agree. Note: T1DM is type 1 diabetes mellitus 

1. Do you support starting a project to improve diabetes control during school hours? ○ Strongly agree ○ Agree ○ Neutral ○ Disagree ○ Strongly disagree

2. Are you open to getting training from professionals on how to support children with T1DM at school? ○ Strongly agree ○ Agree ○ Neutral ○ Disagree ○ Strongly disagree

3. Would you be willing to learn how to help a student with T1DM who isn't feeling well? ○ Strongly agree ○ Agree ○ Neutral ○ Disagree ○ Strongly disagree

4. Are you willing to assist a student with T1DM until an ambulance or specialist care arrives? ○ Strongly agree ○ Agree ○ Neutral ○ Disagree ○ Strongly disagree

5. Are you willing to receive training from professionals regarding school measures for students with other chronic diseases? ○ Strongly agree ○ Agree ○ Neutral ○ Disagree ○ Strongly disagree

Practices: P) Kindly answer each statement below with “Yes”, “No”, OR “Not sure”.

Have you taught a student with T1DM? ○ Yes. Skip to question 1 ○ No. Skip to question 6 of practices ○ Not sure. Skip to question 6 of practices

For teachers who have encountered a student with T1DM: 

1. Have you actively implemented specific measures to ensure the safety and well-being of a diabetic student during regular class hours? ○ Yes ○ No ○ Not sure

2. In emergency situations related to a student with type 1 diabetes's health, have you followed specific protocols or procedures? ○ Yes ○ No ○ Not sure

3. Do you regularly check in with students with type 1 diabetes to ensure they are following their care plan, including checking blood sugar levels or administering insulin if needed? ○ Yes ○ No ○ Not sure

4. Have you received specific training or professional development related to supporting students with type 1 diabetes in your classroom? ○ Yes ○ No ○ Not sure

5. Are you comfortable discussing and addressing the specific needs of a student with type 1 diabetes with their parents or caregivers? ○ Yes ○ No ○ Not sure

For teachers who haven't encountered a student with T1DM:

6. If you have a student with type 1 diabetes in your classroom, do you feel confident in your ability to implement specific measures to ensure their safety and well-being during regular class hours? ○ Yes ○ No ○ Not sure

7. If you were to have a student with type 1 diabetes in your classroom, would you proactively seek information about their specific needs and care requirements? ○ Yes ○ No ○ Not sure

8. In a hypothetical scenario where a student with type 1 diabetes needed to check their blood sugar level or administer insulin during class, do you think you would handle the situation effectively? ○ Yes ○ No ○ Not sure

9. Considering you haven't encountered a student with type 1 diabetes, would you be willing to undergo training or seek guidance on how to support and accommodate their needs in the classroom? ○ Yes ○ No ○ Not sure

10. If you were aware that a student in your class has type 1 diabetes, would you make an effort to communicate with their parents or caregivers to stay informed about their health and care plan? ○ Yes ○ No ○ Not sure

Appendix 2

**Table 10:** Knowledge gaps among school teachers regarding students with T1DM T1DM - type 1 diabetes mellitus

Knowledge questions of the school teachers (N = 402)	Knowledge gaps N (%)
T1DM is diagnosed most frequently during childhood.	204 (50.7%)
T1DM is a chronic disease.	133 (33.0%)
T1DM is caused by poor lifestyle such as lack of physical activity, unhealthy food, pollution, etc.	272 (67.7%)
T1DM can have serious complications if not treated appropriately.	91 (22.6%)
People with T1DM are advised to regularly measure the level of sugar (glucose in their blood).	77 (19.2%)
The normal blood sugar level in children upon awaking is 4-7 mmol/L or equivalent to 72-120 mg/dL.	235 (58.5%)
The level of sugar in the blood is important to determine the dose of treatment to be taken.	74 (10.5%)
If blood sugar levels are normal for 3 months diabetes medications can be stopped.	264 (65.7%)
People with T1DM only use insulin when complications occur.	284 (70.6%)
Insulin use carries a risk of (low blood sugar) hypoglycemic episode.	219 (54.4%)
People with T1DM must eat more carbohydrates (sugars) because their bodies do not produce the sugar needed for energy.	228 (56.7%)
Red meat is a high-carb food.	182 (45.3%)
People who take diabetes medications don't need to care about healthy eating.	103 (25.6%)
Injuries to the skin are more serious in people with T1DM because the risk of infection is higher.	127 (31.6%)
Intense exercise may cause severe (low blood sugar) hypoglycemia in patients with T1DM.	167 (41.5%)
There are no warning signs of (low blood sugar) hypoglycemia.	162 (40.3%)
People with T1DM need regular medical exams for their eyesight, kidney function, and nerve function.	102 (25.4%)
There are no specific measures/precautions for people with T1DM to exercise.	190 (47.3%)
If a person with T1DM feels unwell, the first thing to do is check their blood sugar level.	77 (19.2%)
If a person with T1DM has an episode of hypoglycemia, they should eat sugary foods or drinks.	177 (44.0%)

Appendix 3 

**Table 11:** Bivariate analysis of practice scores with demographics, knowledge, and attitude variables Values represent percentage of participants with good practices within each subgroup. Practices (taught): Practices of teachers who taught students with type 1 diabetes mellitus. Practices (non-taught): Practices of teachers who did not teach students with type 1 diabetes mellitus. T1DM - type 1 diabetes mellitus

Correlation name	Good practices among groups	Statistical test	Significance (p < 0.05)	Findings
Practices (taught) * age	70.8% (20-30), 56.2% (21-40), 63.8% (41-50), 67.6% (>50)	Chi-square	0.593	No significance
Practices (non-taught) * age	54.1% (20-30), 77.0% (21-40), 75.9% (41-50), 83.3% (>50)	Chi-square	0.024	Significant association observed
Practices (taught) * gender	61.8% (males), 64.2% (females)	Chi-square	0.758	No significance
Practices (non-taught) * gender	62.96% (males), 77.05% (females)	Chi-square	0.040	Significant association observed
Practices (taught) * qualification	63.6% (diploma), 65.9% (bachelor), 53.3% (masters), 50.0% (PhD)	Chi-square	0.607	No significance
Practices (non-taught) * qualification	60.0% (diploma), 72.3% (bachelor), 82.6% (masters),72.7% (PhD)	Chi-square	0.324	No significance
Practices (taught) * under and postgraduate	65.7% (under), 53.1% (post)	Chi-square	0.179	No significance
Practices (non-taught) * under and postgraduate	71.2% (under), 80.7% (post)	Chi-square	0.162	No significance
Practices (taught) * scientific vs. non-scientific	58.4% (scientific), 65.6% (non-scientific)	Chi-square	0.368	No significance
Practices (non-taught) * scientific vs. non-scientific	75.0% (scientific), 73.3% (non-scientific)	Chi-square	0.822	No significance
Practices (taught) * professional title	71.4% (principal), 50.0% (deputy principal), 62.9% (teachers), 70.0% (counsellor)	Chi-square	0.752	No significance
Practices (non-taught) * professional title	83.3% (principal), 60.0% (deputy principal), 73.6% (teachers), 75.0% (counsellor)	Chi-square	0.854	No significance
Practices (taught) * type of school	67.8% (public), 59.5% (private)	Chi-square	0.252	No significance
Practices (non-taught) * type of school	69.7% (public), 75.6% (private)	Chi-square	0.339	No significance
Practices (taught) * years of experience	70.8% (0-5), 57.8% (6-10), 62.0% (11-15), 54.8% (16-20), 69.6% (>20)	Chi-square	0.556	No significance
Practices (non-taught) * years of experience	70.3% (0-5), 73.6% (6-10), 66.6% (11-15), 77.4% (16-20), 82.6% (>20)	Chi-square	0.459	No significance
Practices (taught) * education level teaching	90.9% (kindergarten), 66.6% (elementary), 65.7% (middle), 58.0% (high school)	Chi-square	0.172	No significance
Practices (non-taught) * education level teaching	47.3% (kindergarten), 72.8% (elementary), 68.5% (middle), 80.1% (high school)	Chi-square	0.021	Significant association observed
Practices (taught) * Emirate	63.4% (Sharjah), 55.5% (Dubai), 55.0% (Abu Dhabi), 85.7% (Ras Al Khaimah), 81.8% (Ajman), 80.0% (Fujairah), 75.0% (Um Al Quwain)	Chi-square	0.465	No significance
Practices (non-taught) * Emirate	76.8% (Sharjah), 72.0% (Dubai), 75.5% (Abu Dhabi), 60.0% (Ras Al Khaimah), 61.5% (Ajman), 40.0% (Fujairah), 50.0% (Um Al Quwain)	Chi-square	0.455	No significance
Practices (taught) * family history of T1DM	63.9% (yes), 64.3% (no), 50.0% (I don’t know)	Chi-square	0.719	No significance
Practices (non-taught) * family history of T1DM	79.4% (yes), 71.2% (no), 70.6% (I don’t know)	Chi-square	0.434	No significance
Practices (taught) * family history of chronic illnesses	61.4% (yes), 66.1% (no), 75.0% (I don’t know)	Chi-square	0.734	No significance
Practices (non-taught) * family history of chronic illnesses	81.3% (yes), 69.3% (no), 45.4% (I don’t know)	Chi-square	0.013	Significant association observed
Practices (taught) * knowledge	64.9% (good knowledge), 59.1% (poor knowledge)	Chi-square	0.486	No significance
Practices (non-taught) * knowledge	89.1% (good knowledge), 60.9% (poor knowledge)	Chi-square	<0.001	Significant association observed
Practices (taught) * attitudes	35.7% (poor attitudes), 65.8% (good attitudes)	Chi-square	0.025	Significant association observed
Practices (non-taught) * attitudes	36.3% (poor attitudes), 77.7% (good attitudes)	Chi-square	<0.001	Significant association observed

Appendix 4 

**Table 12:** Bivariate analysis of attitude scores with demographics, knowledge, and practice variables Values represent percentages of participants with good attitudes within each subgroup. Practices (taught): Practices of teachers who taught students with type 1 diabetes mellitus. Practices (non-taught): Practices of teachers who did not teach students with type 1 diabetes mellitus. T1DM - type 1 diabetes mellitus

Correlation name	Good attitudes among groups	Statistical test	Significance (p < 0.05 )	Findings
Attitudes * age	86.8% (20-30), 92.6%(31-40), 91.6% (41-50), 90.6% (>50)	Chi-square	0.632	No significance
Attitudes * gender	91.7% (males), 90.7% (females)	Chi-square	0.847	No significance
Attitudes * qualification	88.4% (diploma), 91.2% (bachelor), 94.7% (master), 69.2% (PhD)	Chi-square	0.055	No significance
Attitudes* under and postgraduate	91.2% (under), 91.0% (post)	Chi-square	0.832	No significance
Attitudes * scientific vs. non-scientific	94.8% (scientific), 89.8% (non-scientific)	Chi-square	0.610	No significance
Attitudes * professional title	92.3% (principal), 84.6% (deputy principal), 91.5% (teachers), 87.5% (counsellor)	Chi-square	0.748	No significance
Attitudes * type of school	89.3% (public), 92.1% (private)	Chi-square	0.213	No significance
Attitudes* years of experience	90.9% (0-5), 93.4% (6-10), 94.5% (11-15), 88.7% (16-20), 88.2% (>20)	Chi-square	0.195	No significance
Attitudes * education level teaching	90.0% (kindergarten), 94.7% (elementary), 91.7% (middle), 89.2% (high school)	Chi-square	0.110	No significance
Attitudes * Emirate	89.9% (Sharjah), 90.6% (Dubai), 94.3% (Abu Dhabi), 100.0% (Ras Al Khaimah), 79.1% (Ajman), 100.0% (Fujairah), 100.0% (Um Al Quwain)	Chi-square	0.548	No significance
Attitudes * family history of T1DM	91.4% (yes), 91.6% (no), 84.0% (I don’t know)	Chi-square	0.262	No significance
Attitudes * family history of chronic illnesses	90.0% (yes), 92.6% (no), 86.6% (not sure)	Chi-square	0.019	Significant association observed
Attitudes * have you taught students with a student with T1DM?	92.1% (yes), 91.6% (no), 86.7% (not sure)	Chi-square	0.006	Significant association observed
Attitudes * knowledge	95.3% (good knowledge), 85.0% (poor knowledge)	Chi-square	<0.001	Significant association observed
Attitudes * practices (taught)	86.1% (poor practices), 95.5% (good practices)	Chi-square	0.025	Significance association observed
Attitudes * practices (non-taught)	76.2% (poor practice), 95.1% (good practice)	Chi-square	<0.001	Significant association observed
